# Regulation of Epstein-Barr Virus Life Cycle and Cell Proliferation by Histone H3K27 Methyltransferase EZH2 in Akata Cells

**DOI:** 10.1128/mSphere.00478-18

**Published:** 2018-11-28

**Authors:** Takaya Ichikawa, Yusuke Okuno, Yoshitaka Sato, Fumi Goshima, Hironori Yoshiyama, Teru Kanda, Hiroshi Kimura, Takayuki Murata

**Affiliations:** a Department of Virology, Nagoya University Graduate School of Medicine, Nagoya, Japan; b Center for Advanced Medicine and Clinical Research, Nagoya University Hospital, Nagoya, Japan; c Department of Pediatrics, Nagoya University Graduate School of Medicine, Nagoya, Japan; d Department of Virology and Parasitology, Fujita Health University School of Medicine, Toyoake, Japan; e Department of Microbiology, Shimane University Faculty of Medicine, Izumo, Japan; f Division of Microbiology, Faculty of Medicine, Tohoku Medical and Pharmaceutical University, Sendai, Japan; University of North Carolina, Chapel Hill

**Keywords:** EBV, EZH2, histone methylation

## Abstract

Epigenetic modifications play a pivotal role in the expression of the genes of Epstein-Barr virus (EBV). We found that *de novo* EBV infection of primary B cells caused moderate induction of enhancer of zeste homolog 2 (EZH2), the major histone H3 lysine 27 (K27) methyltransferase. To investigate the role of EZH2, we knocked out the EZH2 gene in EBV-negative Akata cells by the CRISPR/Cas9 system and infected the cells with EBV, followed by selection of EBV-positive cells. During the latent state, growth of EZH2-knockout (KO) cells was significantly slower after infection compared to wild-type controls, despite similar levels of viral gene expression between cell lines. After induction of the lytic cycle by anti-IgG, KO of EZH2 caused notable induction of expression of both latent and lytic viral genes, as well as increases in both viral DNA replication and progeny production. These results demonstrate that EZH2 is crucial for the intricate epigenetic regulation of not only lytic but also latent gene expression in Akata cells.

**IMPORTANCE** The life cycle of EBV is regulated by epigenetic modifications, such as CpG methylation and histone modifications. Here, we found that the expression of EZH2, which encodes a histone H3K27 methyltransferase, was induced by EBV infection; therefore, we generated EZH2-KO cells to investigate the role of EZH2 in EBV-infected Akata B cells. Disruption of EZH2 resulted in increased expression of EBV genes during the lytic phase and, therefore, efficient viral replication and progeny production. Our results shed light on the mechanisms underlying reactivation from an epigenetic point of view and further suggest a role for EZH2 as a form of innate immunity that restricts viral replication in infected cells.

## Abstract (precis)

The life cycle of EBV is regulated by epigenetic modifications, such as CpG methylation and histone modifications. Here, we found that the expression of EZH2, which encodes a histone H3K27 methyltransferase, was induced by EBV infection; therefore, we generated EZH2-KO cells to investigate the role of EZH2 in EBV-infected Akata B cells. Disruption of EZH2 resulted in increased expression of EBV genes during the lytic phase and, therefore, efficient viral replication and progeny production. Our results shed light on the mechanisms underlying reactivation from an epigenetic point of view and further suggest a role for EZH2 as a form of innate immunity that restricts viral replication in infected cells.

## INTRODUCTION

Epstein-Barr virus (EBV) is a human gammaherpesvirus that infects >90% of the population worldwide. EBV is transmitted via saliva, typically from close family members during infancy or childhood. EBV causes infectious mononucleosis and several proliferative disorders of lymphocytes, such as Burkitt lymphoma, Hodgkin’s lymphoma, posttransplant lymphoproliferative disorder (PTLD), chronic active EBV infection (CAEBV), and T/NK cell lymphoma. It is also involved in several epithelial cancers, including gastric carcinoma and nasopharyngeal carcinoma (NPC) (1).

EBV can establish two forms of infection in cells: latent and lytic. During latency, the viral DNA genome exists as episomes in the nucleus, where it expresses a small number of latent viral genes, including EBV nuclear antigens (EBNAs), latent membrane proteins (LMPs), and noncoding RNAs, such as EBV-encoded small RNAs (EBERs) and microRNAs (miRNAs) in the BamHI-A rightward transcripts (BARTs) or the BHRF1 region. The latent mode is further categorized into at least four types according to the pattern of the expressed latent genes. Latent EBV-infected memory B cells express only EBERs (latency type 0). Neoplasms such as Burkitt lymphoma and gastric carcinoma typically express only EBERs and EBNA1 (type I), whereas some types of Hodgkin’s lymphoma, NPC, CAEBV, and T/NK lymphomas, produce EBERs, EBNA1, LMP1, and LMP2 genes (type II). In most cases of PTLD or lymphoblastoid cell lines (LCLs), EBNA2, EBNA3, and EBNA-LP (type III) are expressed in addition to type II genes. Expression of BART miRNAs is higher in latency I and II, whereas higher levels of BHRF1 miRNAs are expressed in latency III (2–4).

During the lytic phase, all EBV genes are coordinately expressed. Induction with chemical or biological reagents such as TPA, HDAC inhibitor, anti-Ig, or TGF-β leads to the expression of two immediate early viral genes, BZLF1 and BRLF1. These genes encode transcriptional activators that enhance transcription of a subset of viral genes categorized as early class genes. Such early genes include viral DNA polymerase catalytic subunit, single-stranded DNA-binding protein (BALF2), and the processivity factor of DNA polymerase (BMRF1), resulting in potent amplification of the EBV DNA genome. Then late class viral genes are produced that encode structural viral proteins, such as gp350/220 and glycoprotein B (gB). With the newly synthesized viral genome and structural proteins, progeny viruses are formed and produced (5, 6).

Virus life cycles are regulated by epigenetic mechanisms of the EBV genome. For example, promoter usage of latent genes is controlled by CpG DNA methylation and histone modifications. Complicated genome structures mediated by the chromatin boundary factor CTCF also play an important role in the regulation of latent genes. Promoters of lytic genes of EBV are associated with both heavy CpG DNA methylation and histone modifications in latency. However, the level of CpG methylation is limited in the BZLF1 promoter (7, 8) and is instead susceptible to silencing by histone modifications (9–12), including trimethylation of histone H3 lysine 27 (H3K27me3). If the BZLF1 promoter were associated with high levels of CpG methylation, the BZLF1 gene would not be immediately expressed, as reversal of CpG methylation would occur too slowly, due to the lack of a demethylation enzyme for CpG methylation in humans. Once expressed, the BZLF1 protein can induce transcription from other heavily CpG-methylated lytic genes, owing to its extraordinary ability to bind to and activate a subset of CpG-methylated viral lytic promoters (13–17).

The significance of CpG methylation by DNA methyltransferases (DNMTs) for EBV gene expression has been clearly demonstrated (18–20); however, the role of histone modification requires further elucidation. EZH2 is the catalytic subunit of polycomb repressive complex 2 (PRC2), which mediates histone H3K27me3 methylation. It plays a crucial role in many physiological or pathological activities, such as cell cycle progression, differentiation, development, senescence, and oncogenesis (21, 22).

We and others have reported that the genome of EBV is modified by histone H3K27me3, and this modification plays a crucial role in the maintenance of latency by silencing the BZLF1 promoter (9, 10). While studies using knockdown methods and small-molecular inhibitors have been informative, they have not ruled out the possibility of unexpected off-target effects. In addition, these studies did not thoroughly examine the effects of histone H3K27me3 on latent viral gene expression. Here, we found that expression of the EZH2 gene, encoding the major methyltransferase of histone H3K27me3, was induced by EBV infection in primary B cells. Comparisons of EBV-infected wild-type (WT) and EZH2-KO cells revealed that this enzyme regulates not only lytic genes but also latent genes such as LMP1 during the lytic state. Interestingly, KO of EZH2 efficiently promoted viral lytic replication and production of progeny virus particles. We also found that growth of EBV-positive cells was attenuated by EZH2 KO. These results indicate that EZH2 plays an important role in Akata cells.

## RESULTS

### Induction of EZH2 by EBV infection.

**FIG 1:**
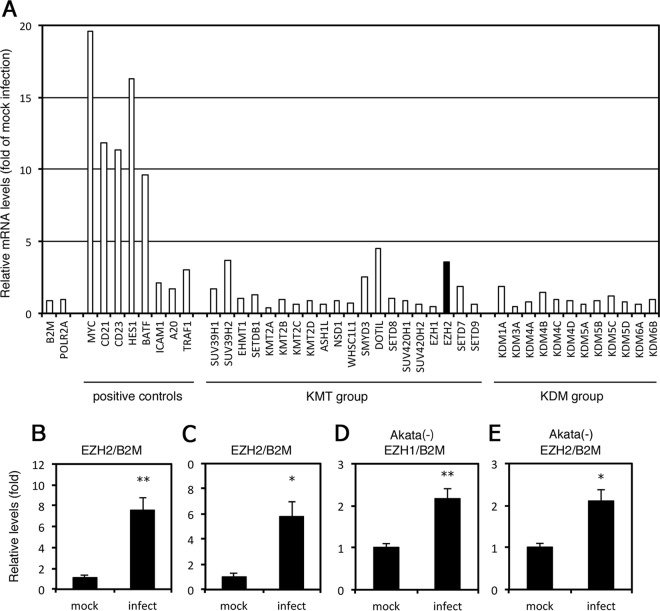
Induction of the EZH2 gene by Epstein-Barr virus (EBV) infection in primary B cells. (A) B cells isolated from peripheral blood mononuclear cells from a healthy donor were sorted using FACSAria II and infected or mock infected with WT EBV at a multiplicity of infection of ∼1. RNA was collected from the infected and mock-infected cells after 2 days. The mRNA was enriched, reverse transcribed, and subjected to RNA sequencing. Relative mRNA levels were calculated according to the frequency per kilobase of exon per million read values after normalization by the values of mock-infected sample. KMT, lysine methyltransferase; KDM, lysine demethylase. The RNA-seq data are available at the DDBJ Sequence Read Archive (accession ID DRA006767). (B and C) Peripheral B cells from different donors were infected with EBV as in panel A and analyzed by qRT-PCR. Relative EZH2 mRNA levels are shown after normalization with beta-2 microglobulin (B2M). Average and SD from three independent infections are shown. Student’s *t* test was performed. (D and E) Akata(−) cells were infected with EBV as in panel A and analyzed by qRT-PCR. Relative EZH1 and EZH2 mRNA levels are shown after normalization with beta-2 microglobulin (B2M). Average and SD from three independent infections are shown. Student’s *t* test was performed. *, *P* < 0.02; **, *P* < 0.002.

In B cell immortalization by EBV, *de novo* EBV infection in primary B cells induces the expression of several cellular genes, such as MYC (23, 24). MYC is an important transcriptional component for viral latency type III and promotion of cell growth (25). To investigate whether the expression of epigenetic modification enzymes is induced by *de novo* EBV infection, we analyzed RNA expression in primary B cells infected with or without the virus by RNA-seq (Fig. 1A). At 2 days after infection, EBV markedly induced expression of MYC, CD21, CD23, HES1, and BATF (Fig. 1A, positive controls) 10- to 20-fold, possibly through EBNA2 as reported previously (23, 24, 26, 27); in contrast, host housekeeping genes including β-2 microglobulin (B2M) and RNA polymerase II (POLR2A) were unaffected. LMP1 expression has been shown to induce several cellular genes, including ICAM1, A20, and TRAF1 (also termed EBI6) (23, 28, 29). Similar results were observed here, with each of these genes exhibiting moderate (2- to 3-fold) induction in response to viral infection (Fig. 1A, positive controls).

Next, we examined lysine methyltransferases (KMTs) and demethylases (KDMs) to assess the role of epigenetic regulation of EBV and the host gene expression, particularly in the context of histone methylations. Although KMTs and KDMs were not strongly induced by EBV infection, the expression of several KMTs was moderately increased, with those of SUV39H2, DOTIL, and EZH2 genes increasing 3.6-, 4.5-, and 3.6-fold, respectively (Fig. 1A, KMT group). The level of EZH2 upregulation might be similar to that of ICAM1 or other LMP1-induced genes.

To confirm the reproducibility, we prepared primary B cells from two other donors and infected them with EBV for 2 days, with expression of the gene validated by quantitative reverse transcription-PCR (qRT-PCR). In these samples, EBV infection resulted in 7.5- and 5.8-fold induction of EZH2 mRNA (Fig. 1B and C, respectively). Taken together, these results suggest that EZH2 may play a significant role in viral and cellular gene expression and immortalization, similar to that of MYC and ICAM1.

　We also examined induction of EZH2, as well as EZH1, in another cell line, Akata(−). Infection of EBV mildly increased mRNA levels of EZH1 and EZH2 (2.2- and 2.1-fold, respectively) (Fig. 1D and E).

### Preparation of EZH2-KO cells.

**FIG 2:**
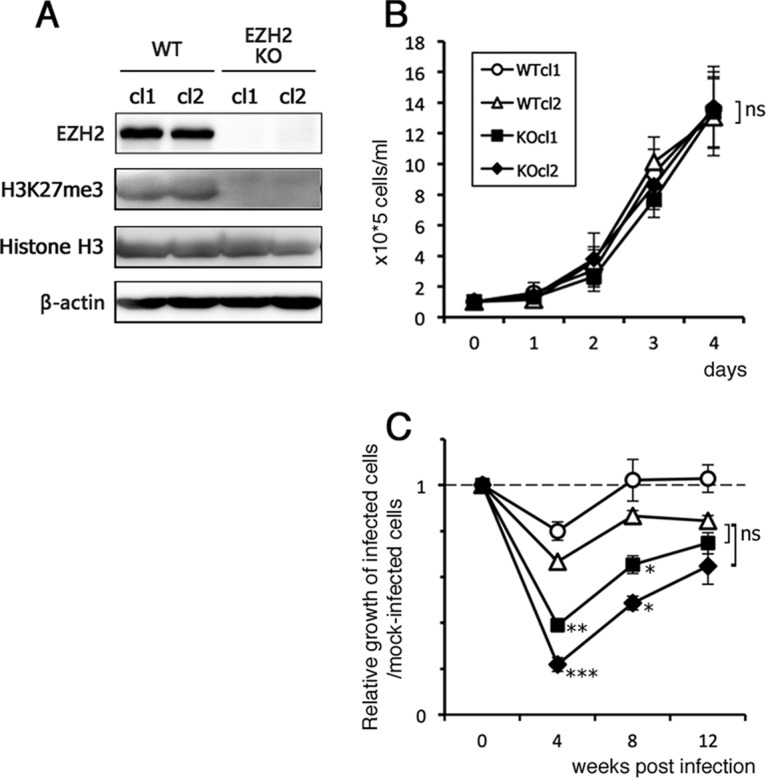
Knockout of EZH2 gene by CRISPR/Cas9 and stable infection of EBV. (A) Loss of EZH2 expression in the EZH2-KO Akata(−) cells. Two independent clones were used (named cl1 and cl2) for both WT and KO. Levels of EZH2, H3K27me3, histone H3, and β-actin in the samples from WT and EZH2-KO cells were examined by Western blotting. (B) Growth kinetics of EZH2-KO Akata(−) cells. A quantity of 1.0 × 10^5^ cells/ml was seeded and cultured. Cell numbers were counted after the indicated number of days. (C) Reduced growth speed in the EZH2-KO cells. The WT and EZH2-KO Akata cells were infected with EBV and cultured in the presence of G418. At 0, 4, 8, and 12 weeks postinfection, cells were seeded at 1.0 × 10^5^ cells/ml, and cell numbers were counted after 3 days. The average and SD from 2 independent replicates are shown as a ratio relative to the value of the starting point (0 week). Student’s *t* test was performed, and asterisks indicate statistical significance (*, *P* < 0.05; **, *P* < 0.01; ***, *P* < 0.005) compared to either of the WT clones. ns, not significant.

To further investigate the importance of EZH2 in the EBV life cycle, we knocked out the EZH2 gene, taking advantage of CRISPR/Cas9 technology to determine the physiological functions of the gene product. Using an EBV-negative Akata cell line, Akata(−), we successfully prepared two EZH2-KO cell lines (cl1 and cl2), in which production of EZH2 was completely disrupted (Fig. 2A). Accordingly, histone trimethylation at H3K27 (H3K27me3) was notably repressed in the KO cells (Fig. 2A). We first compared proliferation of these cell lines before EBV infection and found no evidence of cell growth defects in EZH2-KO cells (Fig. 2B). Next, we infected KO and WT cells with EBV, which was originally produced in AGS/EGFP-EBV cells (30). The virus from AGS/EGFP-EBV cells is a recombinant Akata virus encoding EGFP and the G418 resistance gene. After culturing in the presence of G418, we successfully obtained cells from both WT and EZH2-KO cell lines in which almost 100% of cells emitted GFP fluorescence (not shown).

### Growth property of EBV-positive EZH2-KO cells.

To analyze whether KO of the EZH2 gene affected cell growth, WT and EZH2-KO cells that were latently infected with EBV were seeded at 1 × 10^5^ cells/ml and incubated, and the cell number was counted at 3 days (Fig. 2C). Due to preliminary data that suggested that growth was slower at earlier time points after infection, we analyzed cell growth at several different time points (0, 4, 8, and 12 weeks postinfection). By week 4, growth of EBV-positive EZH2-KO cells was markedly slower than that of WT cells (Fig. 2C); however, beyond this point, growth of EBV-positive WT and KO cells increased (Fig. 2C). Taken together, these data show that the absence of EZH2 had a negative effect on cell growth in response to EBV infection. Based on these results, we speculate that EBV lytic genes might not be adequately silenced in the KO cells and that expression of lytic genes might be stressful for cells. Alternatively, because EBNA3A/C suppresses the expression of tumor suppressor genes, such as BIM, p14^ARF^, and p16^INK4A^, through EZH2 and H3K27me3 (31, 32), KO of EZH2 might result in a decrease of EBNA3A/C function and slow cell growth.

### Expression of viral genes in EZH2-KO cells during latency.

We first analyzed the latency pattern of EBV in WT and KO cells using MutuI and MutuIII cell lines as models of latency I and III, respectively. LMP1 and EBNA2 mRNAs were abundantly expressed in MutuIII, with minimal expression in MutuI, WT, and EZH2-KO cells. In contrast, EBNA1 was highly expressed in all four cell lines (not shown). Based on these results, the EBV-positive cell lines used here most likely represent latency I, consistent with previous results (33).

**FIG 3:**
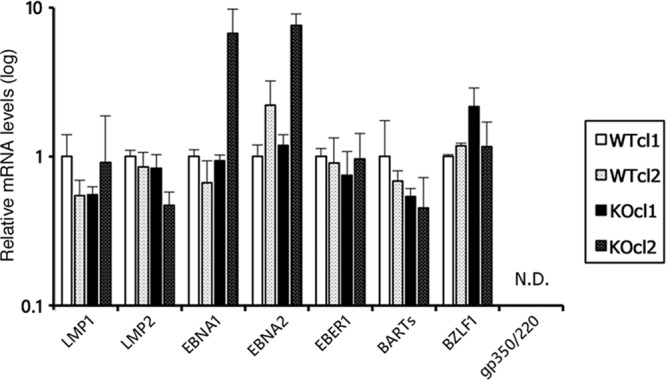
Viral gene expression in WT and EZH2-KO Akata cells during latent infection. RNA was harvested from WT and EZH2-KO cells (two clones each) latently infected with EBV and subjected to qRT-PCR. The data are normalized to the value of β2-microglobulin and shown as fold change for the average and standard deviation from three samples.

We quantified the mRNA levels of both latent and lytic EBV genes expressed during latency (without lytic induction) by qRT-PCR (Fig. 3). Clonal variants were evident between cell lines, with one KO cell line expressing significantly higher levels of EBNA1 and EBNA2 than other lines (Fig. 3, hatched black bars). However, aside from a few notable exceptions, expression levels of viral genes, including BZLF1, were broadly similar between WT and EZH2-KO cells (Fig. 3). This result coincides with our previous data that knockdown of EZH2 or pharmacological inhibition of EZH2 by DZNep failed to induce BZLF1 expression at least in Akata cells (9). Based on these data, EZH2 may not be the major regulator of BZLF1 repression and maintenance of EBV latency in Akata cells. The gp350/220 gene was undetectable (Fig. 3).

### Low histone H3K27me3 levels in EZH2-KO cells during latency.

**FIG 4:**
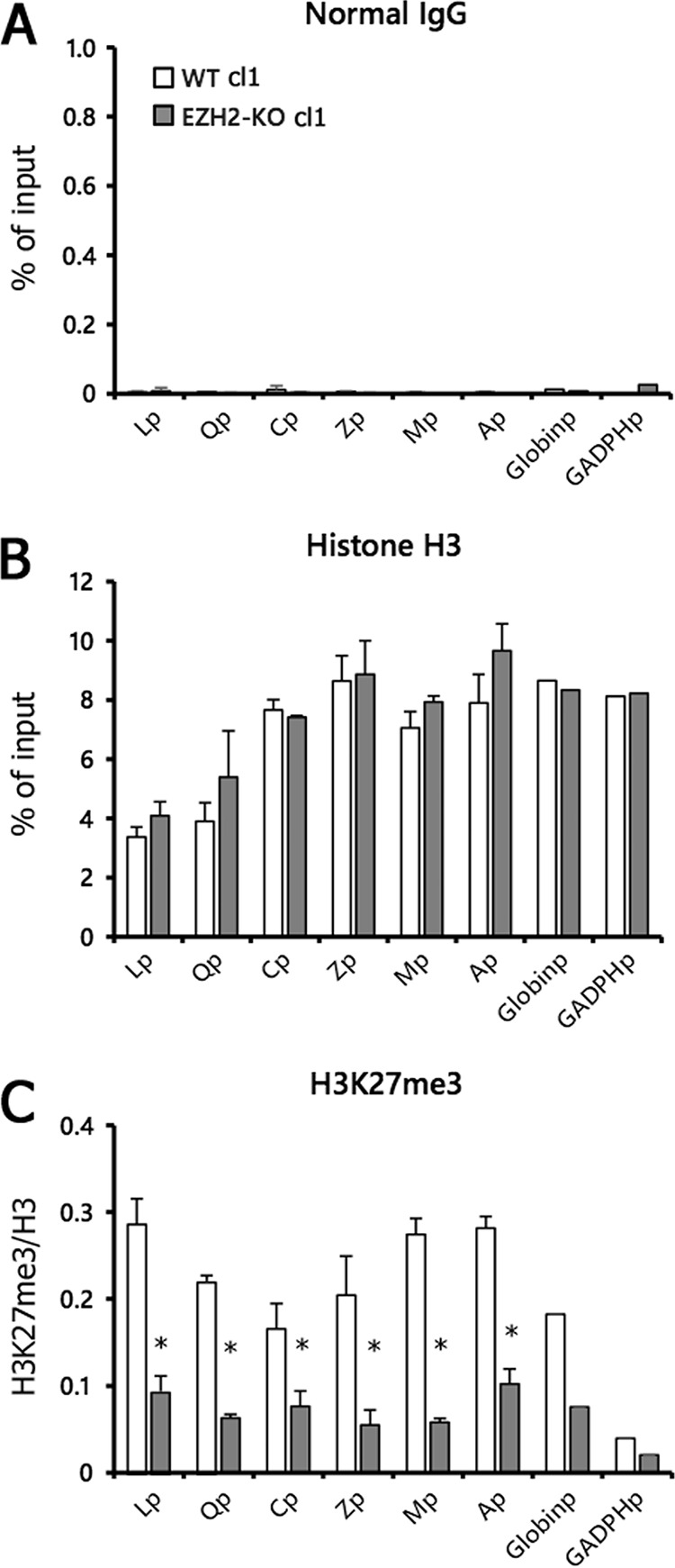
Prominent decrease in H3K27me3 modification in EZH2-KO cells. (A to C) WT and EZH2-KO Akata cells latently infected with EBV (cl1) were subjected to chromatin immunoprecipitation (ChIP) analysis using normal IgG (A), anti-histone H3 (B), or anti-histone H3K27me3 (C) antibodies. Data for normal IgG and histone H3 are shown as the percentage of the input sample. Data for H3K27me3 (C) are shown after normalization to the value of histone H3. Lp, LMP1 promoter; Qp, Q promoter; Cp, C promoter; Zp, BZLF1 promoter; Mp, BMRF1 promoter; Ap, BALF2 promoter; Globinp, globin promoter; GAPDHp, GAPDH promoter. Student’s *t* test was performed. *, *P* < 0.01.

To assess histone H3K27me3 levels in the viral promoters in EZH2-KO cells latently infected with EBV, we carried out ChIP assays followed by qPCR (Fig. 4). We quantified histone levels for the following promoters: LMP1 (Lp), Q (Qp), and Cp latent promoters; Zp (BZLF1 promoter), Mp (BMRF1 promoter), and Ap (BALF2 promoter) lytic promoters; and globin (Globinp) and GAPDH (GAPDHp) host control promoters. When normal IgG was used for precipitation as a negative control, <0.03% of the input DNA was detected (Fig. 4A). Histone H3 levels were almost comparable between WT and EZH2-KO (Fig. 4B), but histone H3K27me3 modification was markedly lower in EZH2-KO (Fig. 4C). While a faint H3K27me3 modification was observed in EZH2-KO cells (Fig. 4C), this residual H3K27 methylation can be explained by the presence of a minor H3K27 methylation enzyme, EZH1 (34). Similar reductions in H3K27me3 modifications were observed in other clones of EZH2-KO cells (cl2) as well (see Fig. 7B).

### Lytic induction caused higher transcription of viral genes in EZH2-KO cells.

**FIG 5:**
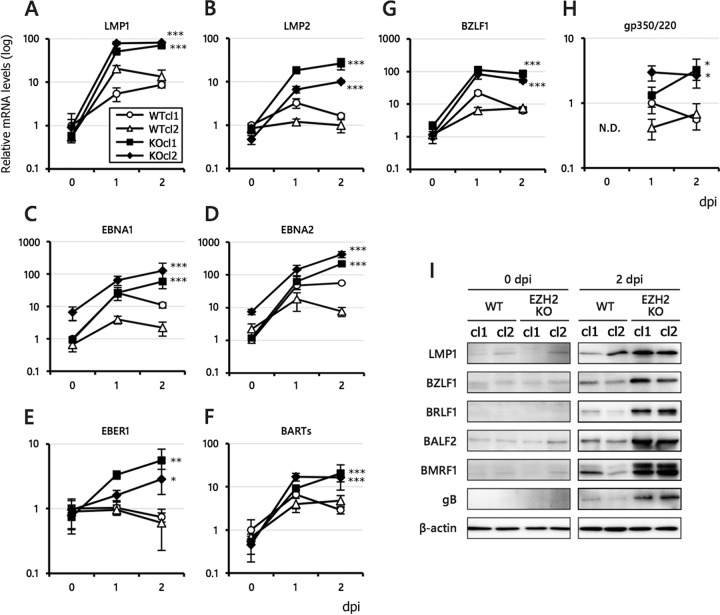
Increased viral gene expression in WT and EZH2-KO Akata cells during lytic phase. (A to H) WT and EZH2-KO cells latently infected with EBV were treated with anti-IgG. RNA was harvested from the cells at 0 (latency), 1, and 2 days postinduction (dpi) and subjected to qRT-PCR analysis. The data are normalized and shown as fold change for the average and SD from three samples. (I) WT and EZH2-KO cells latently infected with EBV were treated with anti-IgG as in panels A to H. Protein samples were collected on days 0 and 2, and LMP1, BZLF1, BRLF1, BALF2, BMRF1, gB, and β-actin levels were assessed by Western blotting. Student’s *t* test was performed, and asterisks indicate statistical significance (*, *P* < 0.05; **, *P* < 0.01; ***, *P* < 0.005) compared to either of the WT clones.

After analyzing the effects of EZH2 disruption on viral gene expression in the latent phase, we analyzed the levels of viral gene expression in KO cells after lytic induction using anti-IgG (Fig. 5). Interestingly, qRT-PCR (Fig. 5A to H) and Western blot (Fig. 5I) analyses revealed that almost all of the EBV genes we examined, including LMPs, EBNAs, EBER1, BARTs, and lytic genes, were significantly higher in the KO cells than in WT after lytic induction. Notably, both the lytic and latent genes responded strongly to EZH2 KO after lytic induction (Fig. 5). These results suggest that EZH2 is involved in the silencing of latent viral genes during the lytic phase.

**FIG 6:**
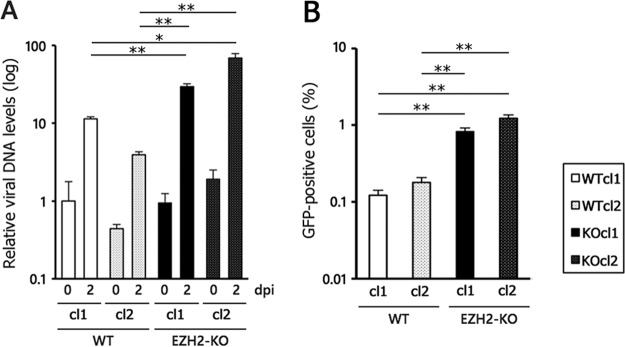
Promotion of the lytic cycle by EZH2-KO in Akata cells. (A) Increased viral DNA replication in EZH2-KO. WT and EZH2-KO cells latently infected with EBV were inoculated with anti-IgG to induce the lytic cycle. Two days after treatment with anti-IgG, the cells were harvested for DNA extraction, and comparisons of viral and host DNA levels were examined by qPCR. The average and SD from 3 independent replicates are shown. The value of the WT day 0 sample (leftmost bar) was set as 1. (B) Progeny virus production was increased by EZH2-KO. The WT and EZH2-KO cells latently infected with EBV were inoculated with anti-IgG to induce the lytic cycle. Two days after treatment with anti-IgG, viral particles present in the medium were harvested and used to infect to Akata(−) cells. As the EBV genome encodes GFP, infected Akata(−) cells become GFP positive. The average and SD of the GFP-positive ratio (%) from 3 independent replicates are shown. Student’s *t* test was performed. *, *P* < 0.001; **, *P* < 0.0005.

Next, because EZH2 KO resulted in higher levels of viral gene expression, we examined whether KO might augment viral lytic DNA replication and progeny production (Fig. 6). Viral DNA levels were significantly higher in EZH2-KO cells infected with EBV relative to WT cells, when induced with anti-IgG (Fig. 6A). Similarly, the progeny production levels were consistent with DNA replication data in that EZH2-KO produced more infectious virions than the WT by about one order of magnitude (Fig. 6B).

### Low histone H3K27me3 levels in EZH2-KO cells in a lytic state.

**FIG 7:**
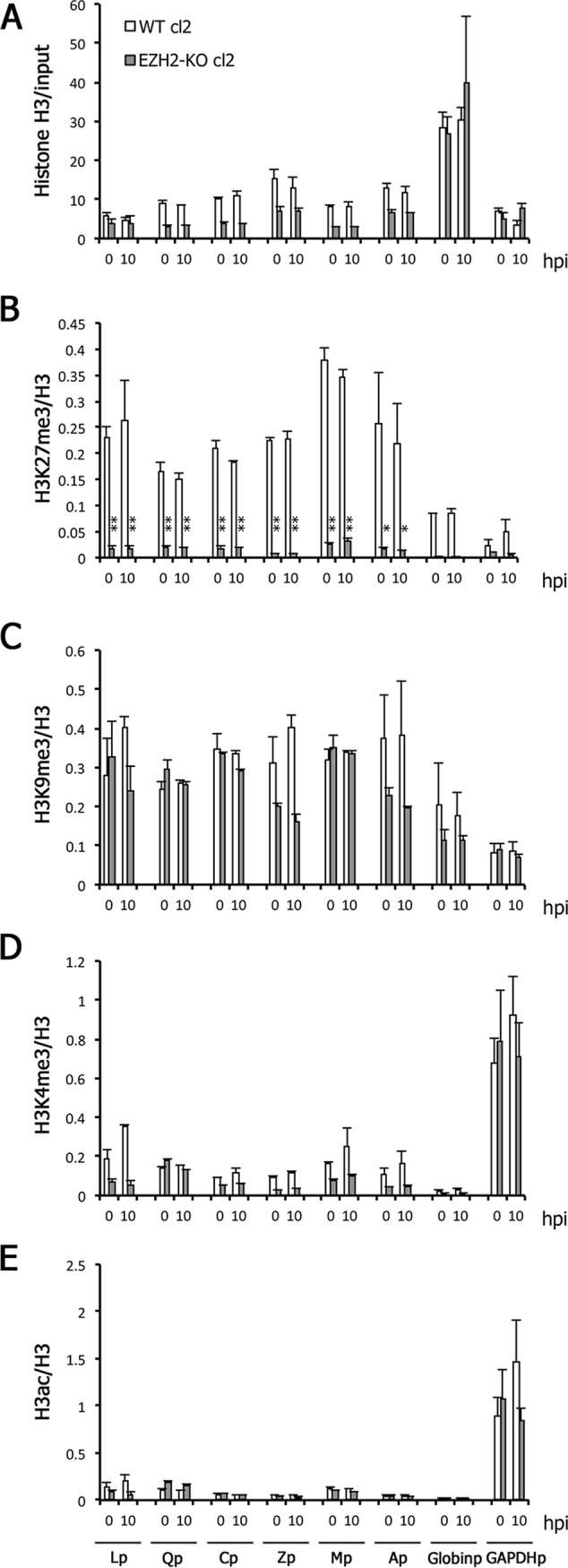
Low H3K27me3 modification in the EZH2-KO cells during reactivation from latency. (A to E) WT and EZH2-KO cells latently infected with EBV (cl2) were treated with anti-IgG. Cells were harvested at 0 (latency) and 10 hpi and analyzed by ChIP using anti-histone H3 (A), anti-histone H3K27me3 (B), anti-histone H3K9me3 (C), anti-histone H3K4me3 (D), or anti-histone H3ac (E) antibodies. Data for histone H3 are shown as the percentage of the input sample (A). The data for other markers (B to E) are shown after normalization to the value of histone H3. Lp, LMP1 promoter; Qp, Q promoter; Cp, C promoter; Zp, BZLF1 promoter; Mp, BMRF1 promoter; Ap, BALF2 promoter; Globinp, Globin promoter; GAPDHp, GAPDH promoter. Student’s *t* test was performed. *, *P* < 0.05; **, *P* < 0.01.

Next, we examined levels of histone H3K27me3 during the lytic phase (Fig. 7). As expected, histone H3K27me3 levels were markedly lower in the viral promoters of EZH2-KO cells than in WT cells (Fig. 7B) even after lytic induction, while levels of total histone H3 were similar or slightly lower in the KO (Fig. 7A). Levels of other negative and active marks, including H3K9me3 (Fig. 7C), H3K4me3 (Fig. 7D), and H3ac (Fig. 7E), were comparable or slightly lower in the viral promoters of the KO cells.

### Effects of pharmacological inhibitor GSK343 on viral gene expression in Akata cells.

**FIG 8:**
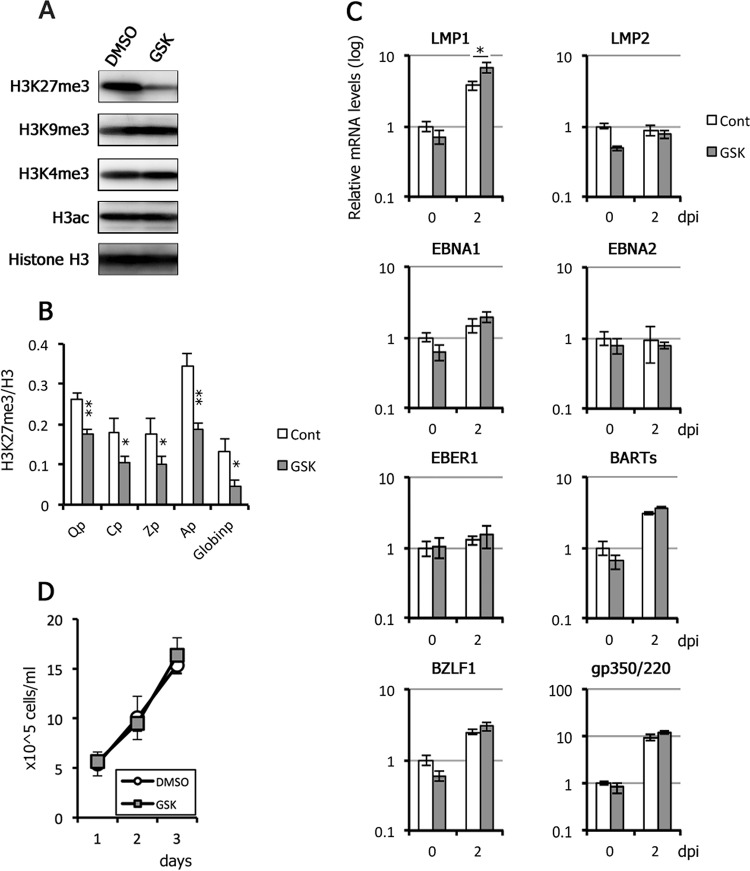
Effect of GSK343, an EZH2 inhibitor, on EBV gene expression in Akata cells. (A) EBV-positive Akata cells were pretreated daily with DMSO (control) or 1 μM GSK343 for 3 days and harvested for Western blotting. (B) EBV-positive Akata cells were treated daily with DMSO (Cont) or 1 μM GSK343 for 3 days and subjected to ChIP assays using anti-histone H3 and -histone H3K27me3 antibodies. The data for H3K27me3 are shown after normalization to the value of histone H3. Qp, Q promoter; Cp, C promoter; Zp, BZLF1 promoter; Ap, BALF2 promoter; Globinp, globin promoter. Student’s *t* test was performed. *, *P* < 0.05; **, *P* < 0.01. (C) After GSK343 treatment for 3 days, Akata cells were then induced with anti-IgG to induce the lytic cycle. RNAs were harvested at 0 and 2 days postinduction (dpi) and subjected to qRT-PCR analysis. The data are normalized and shown as fold change for the average and SD from three samples. Student’s *t* test was performed. *, *P* < 0.02. (D) EBV-positive Akata cells were treated daily with DMSO (control) or 1 μM GSK343, and cell numbers were counted.

To extend our results from Akata KO cells, we examined viral gene expression in WT Akata cells by taking advantage of an EZH2 inhibitor, GSK343 (Fig. 8). EBV-positive Akata cells were pretreated with GSK343 for 3 days due to the delayed response of H3K27me3 levels to inhibition by GSK343. Western blot and ChIP analyses confirmed histone H3K27me3-specific inhibition (Fig. 8A and B). After 3 days of treatment with GSK343, the cells were treated with anti-IgG on day 0 to induce the EBV lytic cycle. RNA was harvested at 0 and 2 days postinduction (dpi) and examined by qRT-PCR. qRT-PCR analyses did not reveal any obvious induction of viral genes by GSK343 alone, or in combination with lytic induction, although lytic genes (BZLF1 and gp350/220), LMP1, EBNA1, and BARTs were induced by anti-IgG to some extent (Fig. 8C). We also confirmed that cell growth was not affected by the inhibitor (Fig. 8D). Although this result seemingly contradicts some of the previous data presented here (Fig. 5), in which many latent and lytic genes were influenced by EZH2 knockout, we speculate that differences in method of inactivation of EZH2 may account for these seemingly paradoxical results; GSK343 may not be able to perfectly inhibit the EZH2 activity.

### Effects of GSK343 on viral gene expression in LCLs.

**FIG 9:**
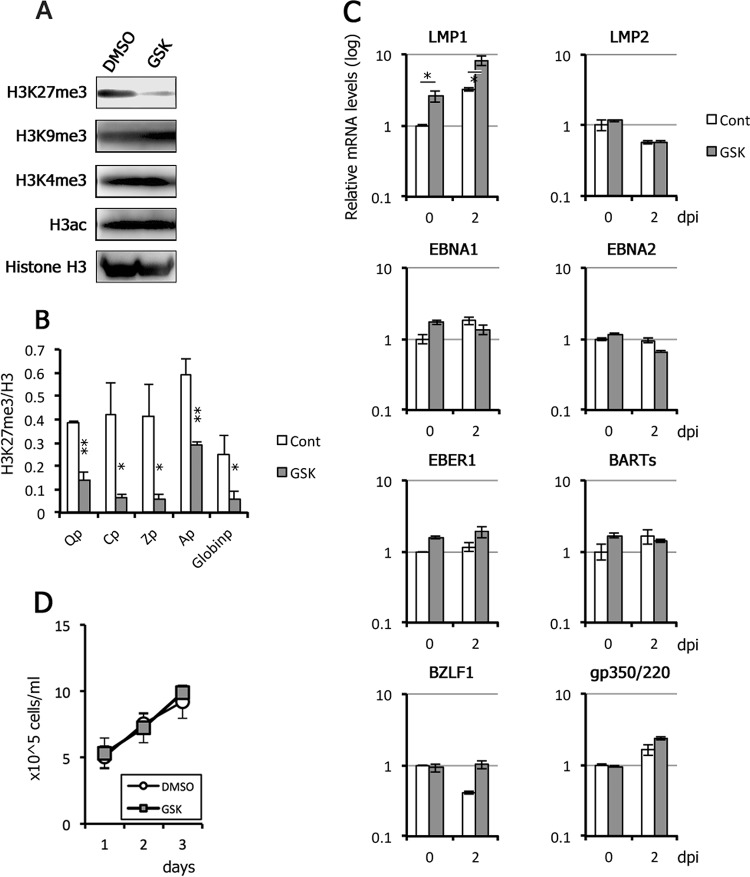
Effect of GSK343 on EBV gene expression in LCLs. (A) LCLs were pretreated daily with DMSO (control) or 1 μM GSK343 for 3 days and harvested for Western blotting. (B) LCLs were treated daily with DMSO (Cont) or 1 μM GSK343 for 3 days and subjected to ChIP assays using anti-histone H3 and -histone H3K27me3 antibodies. The data for H3K27me3 are shown after normalization to the value of histone H3. Qp, Q promoter; Cp, C promoter; Zp, BZLF1 promoter; Ap, BALF2 promoter; Globinp, globin promoter. Student’s *t* test was performed. *, *P* < 0.05; **, *P* < 0.01. (C) LCLs with or without GSK343 treatment were then supplemented with TPA (50 ng/ml), A23187 (200 nM), and sodium butyrate (5 mM) in order to induce the lytic cycle. RNAs were harvested at 0 and 2 days postinduction (dpi) and subjected to qRT-PCR analysis. The data are normalized and shown as fold change for the average and SD from three samples. Student’s *t* test was performed. *, *P* < 0.005. (D) LCLs were treated daily with DMSO (control) or 1 μM GSK343, and cell numbers were counted.

Next, we examined viral gene expression in LCLs, too, after GSK343 treatment (Fig. 9). LCLs were pretreated with GSK343 for 3 days. Western blot and ChIP analyses confirmed inhibition of H3K27me3 (Fig. 9A and B). After 3 days of treatment with GSK343, the cells were treated with TPA, A23187 (calcium ionophore), and sodium butyrate on day 0 to induce the EBV lytic cycle. RNA was harvested at 0 and 2 days postinduction (dpi) and examined by qRT-PCR. The result was quite similar to that of Akata cells (Fig. 8), in that GSK343 did not markedly induce viral gene expression even after lytic induction (Fig. 9C). The cell growth was comparable with or without GSK343 (Fig. 9D).

It is noteworthy that expression of LMP1, a latent gene, was increased 2.6-fold in response to GSK343 treatment before lytic induction, with expression further enhanced 8.3-fold in response to lytic induction (Fig. 9C, LMP1). In Akata cells, LMP1 was increased to 3.8-fold by anti-IgG treatment, and the level further increased to 6.7-fold by GSK343 (Fig. 8C, LMP1). Because LMP1 transcription was also significantly activated by EZH2 KO (Fig. 5A), these data suggest that LMP1 may be more susceptible to silencing by EZH2, especially in the lytic state.

## DISCUSSION

This study shows that EZH2, the major histone H3K27me3 methyltransferase, plays a crucial role in the regulation of both lytic and latent gene expression, at least in Akata cells. We also showed that EZH2 is important for promoting cell growth in Akata cells.

The expression level of EZH2 increased upon infection of primary B cells by EBV, suggesting its importance in virus function (Fig. 1). Induction of MYC peaked at 19.6-fold 2 days after infection, while induction of ICAM and EZH2 was only 2.1- and 3.6-fold, respectively (Fig. 1A). It is speculated that this difference in induction is dependent on the EBV gene responsible for the induction; MYC is induced by EBNA2 (24), which is abundantly expressed immediately after *de novo* infection, and ICAM1 expression is mediated through NF-κB activation by LMP1 (23), which is less abundant for several days after infection in primary B cells (35). Like ICAM1, the EZH2 gene may also be induced by the activation of NF-κB from LMP1, because NF-κB activation has been reported to induce EZH2 gene expression (36, 37).

We prepared and analyzed the EZH2-KO cell lines derived from an EBV-negative Burkitt lymphoma B cell line, Akata(−) (Fig. 2 to 7). In addition, we prepared KO cells from HEK293 cells, but unexpectedly, the disruption of EZH2 in HEK293 had little or no effect on the life cycle of EBV (not shown). It remains unclear why the effects of EZH2 on EBV gene expression appear to be more explicit in B cells. It is possible that other suppressive histone-modifying enzymes might play a dominant role in HEK293. For example, EZH1, rather than EZH2, might be more important for histone H3K27 methylation in HEK293.

We observed low histone H3K27me3 modification in both the host and viral genomes in the EZH2-KO cells (Fig. 4), but these changes did not result in differences in viral gene expression during latency (Fig. 3). Therefore, it is likely that H3K27me3 is not the only determinant of the observed expression pattern in latent cells, and other epigenetic modifications also play roles in regulation of EBV gene transcription in Akata cells.

Unlike comparable gene expression observed in EZH2-KO cells during latency (Fig. 3), lytic induction by anti-IgG treatment caused potent global induction of EBV genes (Fig. 5), which was correlated with the ubiquitous decrease in H3K27me3 (Fig. 7). We do not know why the effect of the EZH2 KO was more emphasized during the lytic cycle, but we speculate that the viral genome may be more active in terms of epigenetic situation during the lytic state, which can have more influence on the KO cells. In addition, the EBV genome can replicate during lytic replication, and thus, it is possible that the newly synthesized viral genome DNA can be regulated by EZH2 more efficiently.

In addition to EBV, other herpesviruses utilize H3K27 methylation for suppression of lytic genes. For example, PRC proteins mediate repressive H2K27 methylation to maintain the latency of herpes simplex virus (38, 39). The lytic cycles of Kaposi sarcoma-associated herpesvirus and human cytomegalovirus are also restricted by PRC proteins (40–42). Here, we showed that EZH2 KO increased expression of lytic and latent genes, as well as viral multiplication (Fig. 5 and 6). The data presented here suggest that EZH2 may act as a part of the innate immune system due to its ability to neutralize or restrict exogenous DNA, particularly that of DNA viruses and retroviruses. However, herpesviruses like EBV have evolved ways of avoiding detection through use of viral latency. Mechanisms of viral latency have even evolved to exploit host silencing systems as a means of avoiding immune detection. Similar mechanisms are seen here, with WT cells exhibiting less damage than EZH2-KO when infected with EBV (Fig. 2C).

Our results indicate that EZH2 and histone H3K27 methylation are profoundly involved in the regulation of viral latent and lytic genes in reactivation. Treatment with pharmacological inhibitors of EZH2 may represent a promising clinical application for the treatment of various cancers (22, 43); however, such an approach may not be appropriate for EBV-positive cancers, as EZH2-KO caused increased viral replication and progeny production (Fig. 5 and 6), which may exacerbate viral transmission and disease burden.

## MATERIALS AND METHODS

### Cells and reagents.

Peripheral blood mononuclear cells (PBMCs), primary B cells, LCLs, Akata(−) cells (44), and AGS/EGFP-EBV cells (30) were cultured in RPMI 1640 medium (Sigma-Aldrich) supplemented with 10% fetal bovine serum and antibiotics. HEK293 EBV-BAC cells were maintained in DMEM (Sigma-Aldrich) supplemented with 10% fetal bovine serum and antibiotics. Antibodies against histone H3 and histone H4 were purchased from Abcam. Antibodies against EZH2, histone H3K27me3, H4K20me3, and H3K9ac were from Active Motif. Anti-histone H3K4me3 and β-actin antibodies were from Millipore and Sigma-Aldrich, respectively. Other antibodies for viral proteins were previously described (45).

### RNA sequence analysis.

PBMCs from EBV-negative healthy donors were prepared, and CD19-positive, CD56-negative, and CD3-negative primary B cells were sorted by FACSAria II (BD Biosciences). EBV was prepared from HEK293 EBV-BAC WT and concentrated by ultracentrifugation, followed by titration. Primary B cells were mock infected or infected with EBV and incubated. After 2 days, RNA was isolated using the RNeasy minikit (Qiagen). Poly(A) RNA enrichment was performed using the Poly(A) RNA Magnetic Isolation Module (NEB), with the resulting mRNA samples subjected to RNA sequencing and data analysis. Sequencing libraries were prepared using an NEBNext Ultra RNA Prep kit for Illumina (NEB), according to the manufacturer’s instructions (46), and sequenced on a HiSeq 2500 next-generation sequencer (Illumina). Data were processed using TopHat and Cufflinks (47, 48) to obtain gene expression profiles expressed as fragments per kilobase of exon per million mapped reads (FPKM).

### KO of EZH2 gene by CRISPR/Cas9.

The oligonucleotides corresponding to the RNA sequence for the human EZH2 gene (CACCGCAATGAGCTCACAGAAGTC and AAACGACTTCTGTGAGCTCATTGC) were hybridized and inserted into the BbsI site of pX330 (Addgene). The resulting pX330-EZH2 plasmid vector was electroporated into an EBV-negative cell line, Akata(−), using the Neon transfection system (Thermo Fisher Scientific). Then the transfected cells were subjected to limiting dilution. The EZH2 gene in the isolated cells was confirmed by PCR and sequencing using the primers TGATTGTTAGTTTGCTGCGG and GAGTATGTTTAGTTCCAATC. We first isolated a cell line in which one of the alleles gained a frameshift mutation [EZH2 (−/+)]. The cell line was again transfected with the pX330-EZH2 vector and subjected to limiting dilution, cloning, and sequencing. Finally, EZH2KO cells were obtained [EZH2 (−/−)]. Off-target clones were used as WT. EBV of the Akata strain with G418 resistance and GFP genes was produced from AGS/EGFP-EBV cells and infected the WT and EZH2-KO Akata(−) cells. Two clones were used for WT and EZH2KO cells (cl1 and cl2).

### Western blotting, quantitative reverse transcription (qRT)-PCR, and ChIP assays.

Western blot analyses were carried out as reported previously (49). The qRT-PCR analyses for LMP1, LMP2, EBNA1, EBNA2, EBER1, BARTs, BZLF1, and gp350/220 genes were carried out using a multiplex real-time PCR system (50). ChIP assays were performed as reported previously (9), using the antibodies described above.

### Accession number(s).

The RNA-seq data are available at DDBJ Sequence Read Archive (accession ID DRA006767).
